# Macrophage Migration Inhibitory Factor (MIF) Prevents Maternal Death, but Contributes to Poor Fetal Outcome During Congenital Toxoplasmosis

**DOI:** 10.3389/fmicb.2018.00906

**Published:** 2018-05-08

**Authors:** Angelica O. Gomes, Bellisa F. Barbosa, Priscila S. Franco, Mayara Ribeiro, Rafaela J. Silva, Paula S. G. Gois, Karine C. Almeida, Mariana B. Angeloni, Andressa S. Castro, Pâmela M. Guirelli, João V. Cândido, Javier E. L. Chica, Neide M. Silva, Tiago W. P. Mineo, José R. Mineo, Eloisa A. V. Ferro

**Affiliations:** ^1^Institute of Natural and Biological Sciences, Federal University of Triângulo Mineiro, Uberaba, Brazil; ^2^Laboratory of Immunophysiology of Reproduction, Institute of Biomedical Science, Federal University of Uberlândia, Uberlândia, Brazil; ^3^Laboratory of Immunopathology, Institute of Biomedical Sciences, Federal University of Uberlândia, Uberlândia, Brazil; ^4^Laboratory of Immunoparasitology, Institute of Biomedical Science, Federal University of Uberlândia, Uberlândia, Brazil

**Keywords:** *Toxoplasma gondii*, maternal-fetal interface, MIF, CD74, IDO, immune response

## Abstract

Migration inhibitory factor (MIF) is a pro-inflammatory cytokine that plays important roles in physiology, pathology, immunology and parasitology, including the control of infection by protozoa parasites such as *Toxoplasma gondii*. As the MIF function in congenital toxoplasmosis is not fully elucidated yet, the present study brings new insights for *T. gondii* infection in the absence of MIF based on pregnant C57BL/6MIF^-/-^ mouse models. Pregnant C57BL/6MIF^-/-^ and C57BL/6WT mice were infected with 05 cysts of *T. gondii* (ME49 strain) on the first day of pregnancy (dop) and were euthanized at 8 dop. Non-pregnant and non-infected females were used as control. Our results demonstrated that MIF^-/-^ mice have more accentuated change in body weight and succumbed to infection first than their WT counterparts. Otherwise, pregnancy outcome was less destructive in MIF^-/-^ mice compared to WT ones, and the former had an increase in the mast cell recruitment and IDO expression and consequently presented less inflammatory cytokine production. Also, MIF receptor (CD74) was upregulated in MIF^-/-^ mice, indicating that a compensatory mechanism may be required in this model of study. The global absence of MIF was associated with attenuation of pathology in congenital toxoplasmosis, but resulted in female death probably because of uncontrolled infection. Altogether, ours results demonstrated that part of the immune response that protects a pregnant female from *T. gondii* infection, favors fetal damage.

## Introduction

The maternal–fetal interface is composed of the placenta, in which trophoblast cells derived from the fetus are in direct contact with the maternal tissues. In mammals with hemochorial placentas (mice and humans, for example), the maternal–fetal interface consists of syncytiotrophoblasts, which are bathed in maternal blood, and extravillous trophoblasts that anchor the placenta in the uterine implantation site, where they are in communication with immune cells from the mother (Moffett and Loke, 2006; Robbins et al., 2012). Some cell types at the maternal fetal interface, such as mast cells (MCs), are under influence of hormonal stimulation, being important cell type able to contribute to Th1/Th2 switch that takes place during pregnancy (Woidacki et al., 2014). This immunological homeostasis is critically important in preventing fetal rejection, and several mechanisms are associated with this immunotolerance, such as the ability of the tryptophan-degrading enzyme indoleamine 2,3-dioxygenase (IDO) to inhibit T-cell proliferation (Sedlmayr, 2007). This tightly regulated maternal immune system at the maternal–fetal interface is important for successful pregnancy but is also associated with susceptibility to infection by pathogens whose infection control requires a predominant Th1 response (Zeldovich and Bakardjiev, 2012).

*Toxoplasma gondii* is a protozoan parasite that can overcome the placental barrier; once the placenta is transposed, congenital toxoplasmosis can occur, leading to spontaneous abortion, preterm labor, or serious diseases in neonates, such as chorioretinitis, intracranial calcifications, and hydrocephalus, the classic triad of congenital toxoplasmosis (Robbins et al., 2012). The symptoms of the disease change according to gestational age: first-trimester maternal infection is associated with the most severe manifestations of the disease (Loke, 1982). The gravity of the disease also depends on the *T. gondii* strain that caused the infection. The Type II strain has been reported as the most prevalent genotype in human congenital toxoplasmosis. The Type I and recombinant strains are usually associated with the most serious cases of the disease (Ajzenberg et al., 2002; Boughattas et al., 2010; Franco et al., 2011, 2015). Despite the importance of this disease, the current treatment strategy is ineffective (Gilbert et al., 2001); little is known about the mechanisms associated with vertical transmission (Robbins et al., 2012) and many people worldwide suffer from the consequences of congenital toxoplasmosis (Guerina et al., 1994; Thiébaut et al., 2007; Carellos et al., 2017).

Previous studies have reported the importance of macrophage migration inhibitory factor (MIF) in congenital (Ferro et al., 2008; de Oliveira Gomes et al., 2011) and acquired (Flores et al., 2008; Terrazas et al., 2010) toxoplasmosis. MIF is a multifunctional proinflammatory cytokine that was first described as a factor produced by lymphocytes associated with inhibition of random macrophage migration during delayed hypersensitivity responses (Bloom and Bennett, 1966; David, 1966). Concerning pregnancy, MIF was described as a factor produced at implantation sites and is mostly expressed by trophoblast cells, presenting a putative role in pregnancy physiology (Bevilacqua et al., 2014). An earlier study by our group, using human placental explants, demonstrated that MIF and its receptor (CD74) were upregulated in response to *T. gondii* infection, thus playing an important role in controlling infection in a gestational age-dependent manner (de Oliveira Gomes et al., 2011). On the other hand, MIF has been associated with pathology in autoimmune diabetes mellitus (Stosic-Grujicic et al., 2008) and cancer (Wilson et al., 2005; O’Reilly et al., 2016), as well as intestinal pathology in *T. gondii* oral infection (Cavalcanti et al., 2011). The pathology is linked with MIF due to its inflammatory properties in addition to its ability to induce cell proliferation and inhibit apoptosis (Wilson et al., 2005; Stosic-Grujicic et al., 2008; Cavalcanti et al., 2011). Moreover, elevated concentrations of MIF in the placenta from malaria positive women were associated with risk of adverse birth outcome, such as, stillbirth and low birth weight deliveries (Singh et al., 2012).

Current research has been focused on methodologies that can counteract MIF activities as a therapeutic approach. For this purpose, neutralizing anti-MIF antibodies and molecules that are capable of neutralizing MIF tautomerase activity have been employed (O’Reilly et al., 2016). However, the consequences of a global absence of MIF have not yet been investigated in a model of congenital toxoplasmosis. Therefore, on the basis of the potential role of MIF in the control of *T. gondii* proliferation and in the putative role of *T. gondii* in pregnancy physiology, the present study aimed to understand the role of MIF in congenital toxoplasmosis based on an MIF knockout mouse model.

## Results

### MIF^-/-^ Mice Lose More Body Weight and Succumb to *T. gondii* Infection Sooner Than Their WT Counterparts

**FIGURE 1:**
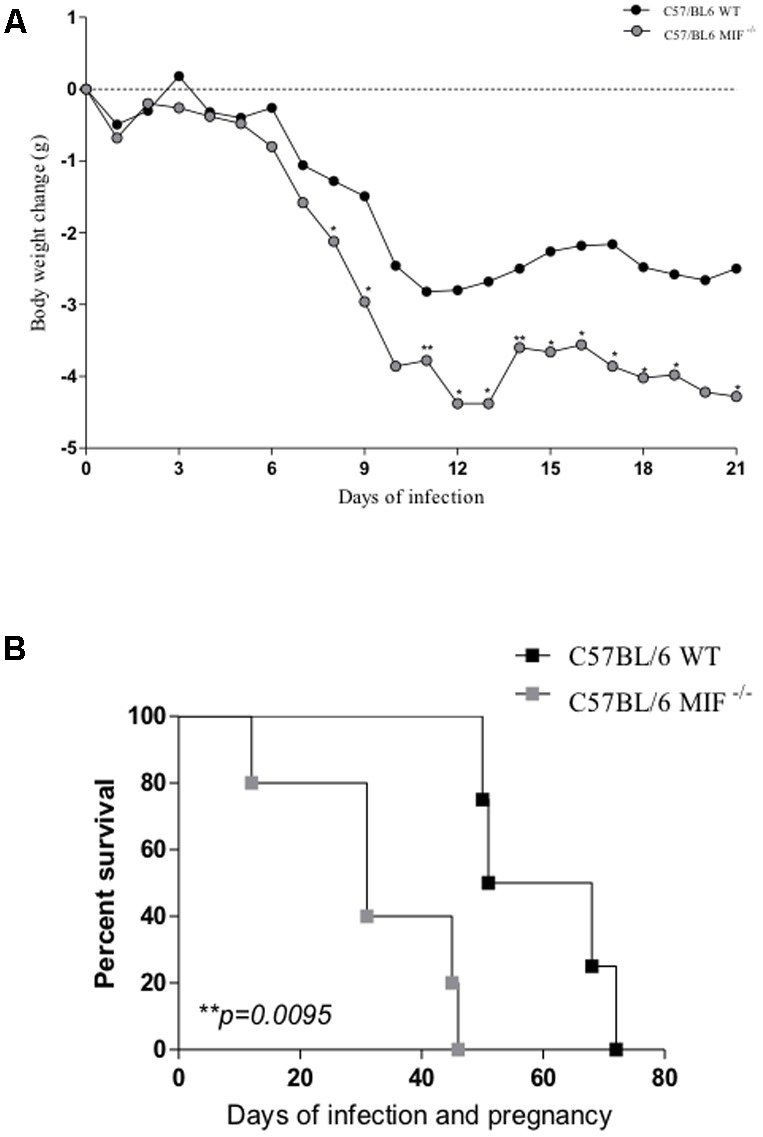
**(A)** Change in body weight in C57BL/6 MIF^-/-^ and C57BL/6 WT mice orally infected with 10 cysts of strain ME49 of *Toxoplasma gondii.* Animals were monitored for 21 days, and the results are presented as the mean of data obtained from 5 animals in each group. Asterisks indicate significant differences between MIF^-/-^ and WT mice (Mann–Whitney test; ^∗^*P <* 0.05; ^∗∗^*P <* 0.01). **(B)** Kaplan–Meier survival curve of infected/pregnant mice. MIF^-/-^ and WT females (*n* = 5 animals per group) were orally infected with five cysts of *T. gondii* (ME49 strain) on the first day of pregnancy and monitored for 80 days after infection. Data were analyzed with a log-rank test. Differences were considered significant if *P <* 0.05 (^∗^).

Non-pregnant MIF^-/-^ and WT females infected with 10 cysts of the ME-49 strain were followed for 21 days for analysis of body weight changes. Our results demonstrated that non-pregnant MIF^-/-^ females had more accentuated change in the body weight from the eighth day onward than WT mice (*P* < 0.01) (**Figure 1A**). Also, pregnant MIF^-/-^ and WT females infected with five cysts of strain ME-49 were followed for 80 days to evaluate the mortality index. We found that pregnant MIF^-/-^ females succumbed to infection before their WT counterparts (*P* = 0.0095) (**Figure 1B**). Also, we detected that none mice gave birth at this time, probably because all mice had abortion during this period.

### The Absence of MIF Is Associated With an Attenuation of Pathology in Congenital Toxoplasmosis

We analyzed the pregnancy outcome in *T. gondii*-infected and non-infected mice from both MIF^-/-^ and WT groups. Our results demonstrated that the percentage of females with successful pregnancy (number of females with implantation sites divided by the number of females with vaginal plug detection) was similar between infected MIF^-/-^ (66%) and WT (60%) females but lower than the non-infected controls of both genetic backgrounds, of which 100% of the females had implantation sites and the presence of previously detected vaginal plugs (**Table 1**).

**Table 1:** Pregnancy outcomes of C57BL/6 MIF^-/-^ and WT mice orally infected with five cysts of *T. gondii* (ME-49 strain) on the first day of pregnancy (day of vaginal plug detection).

Implantation sites	Experimental Groups			
	C57BL/6 WT		C57BL/6 MIF ko	
	(P/NI)	(P/I)	(P/NI)	(P/I)
Number of females with implantation sites/number of females with vaginal plug detection	5/5 (100%)	6/10 (60%)	5/5 (100%)	6/9 (66%)
Average of number of implantation sites^∗^	8.0 (100% N)	4.6 (48%N + 52%R)	7.8 (100% N)	4.8 (66%N + 34%R)

*Females were euthanized at 8 days of pregnancy/infection. The uterus was macroscopically examined with a focus on the features of implantation sites. ^∗^The average of total implantation sites was calculated as the ratio of total implantation sites [normal sites (N) plus reabsorption sites (R)] for each group/number of total females of the group. The fetal reabsorption sites were identified by their small size and necrotic, hemorrhagic appearance, compared with normal implantation sites. The percentage of normal and reabsorption sites related to total implantation site per group is indicated in parenthesis. P/NI, pregnant/non-infected; P/I, pregnant/infected.*

Regarding the average number of implantation sites, we found similar results between infected WT (4.6) and MIF^-/-^ (4.8) females. Of these, 48 and 66% were considered normal implantation sites for WT and MIF^-/-^ females, respectively, whereas 52 and 34% were considered reabsorption sites, respectively. Likewise, in non-infected controls, we observed similar averages of implantation sites for WT (8.0) and MIF^-/-^ (7.8) females, but all were considered normal implantation sites, with no reabsorption site (**Table 1**).

**FIGURE 2:**
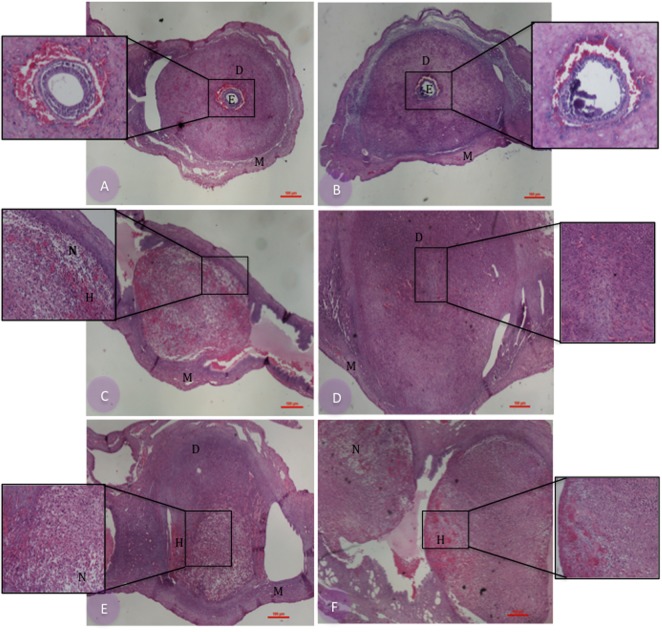
Histopathological analysis of implantation sites from pregnant C57BL/6 MIF^-/-^ and C57BL/6 WT mice infected or not by *Toxoplasma gondii.* Paraffin-embedded 4-μm tissue sections were stained with hematoxylin-eosin. Pregnant/non-infected WT **(A)** and MIF^-/-^ **(B)** controls; pregnant/infected WT **(C,E)** and MIF^-/-^ **(D,F)** females. E, embryo; D, decidual zone; M, myometrium; N, decidual cells in necrosis; H, hemorrhagic areas. Bar scale = 100 μM. The inserts in the figures show increased magnification of the selected area (original magnification in the insert is 10x).

Histopathological analysis of the implantation sites demonstrated that non-infected controls presented normal morphology in both WT and MIF^-/-^ mice, in which the decidual zones, myometrium, and embryos were preserved (**Figures 2A,B**, respectively). In infected females, however, we detected both normal and pathologic implantation sites for both genetic backgrounds, differing mostly in the frequency of pathologic features found in each group (**Figures 2C–F**). Five of six infected WT females presented necrotic implantation sites, and four of these were also hemorrhagic. Apoptotic cells were also present in the decidua of these implantation sites (**Figures 2C,E**). Concerning MIF^-/-^-infected females, three of six of them presented normal implantation sites, although the embryos were not visualized in these tissue sections (**Figure 2D**). The other three presented pathologic implantation sites, one exhibiting an implantation site with edema and two others presenting necrotic and hemorrhagic implantation sites in addition to apoptotic bodies (**Figure 2F**).

### WT Mice Secrete Higher Levels of Proinflammatory Cytokines Than MIF^-/-^ Mice

**FIGURE 3:**
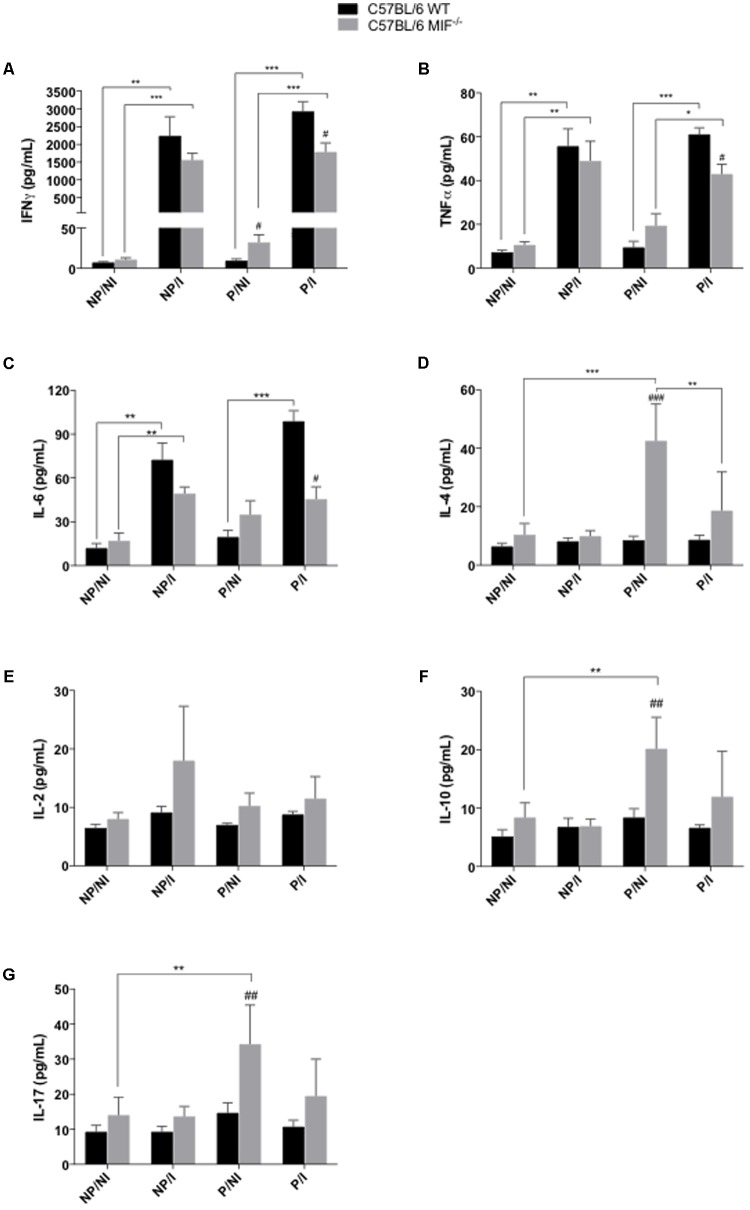
Cytometric bead assay (CBA) for cytokine measurements in serum samples of pregnant and non-pregnant C57BL/6 MIF^-/-^ and C57BL/6 WT mice orally infected by *Toxoplasma gondii.* Serum samples were collected within 8 days of infection/pregnancy. Non-infected pregnant and non-pregnant mice were used as controls. Data indicate levels of IFN-γ **(A)**, TNF-α **(B)**, IL-6 **(C)**, IL-4 **(D)**, IL-2 **(E)**, IL-10 **(F)**, and IL-17 **(G)**, presented as mean and SEM from serum samples of five animals per group and are expressed in pg/mL. ^#^Significant differences between MIF^-/-^ and WT mice in the same experimental condition (^#^*P <* 0.05, ^##^*P <* 0.01, ^###^*P <* 0.001). ^∗^Comparisons between multiple experimental conditions within the same group (NP/NI, non-pregnant/non-infected; NP/I, non-pregnant/infected; P/NI, pregnant/non-infected; P/I, pregnant/infected) are indicated by bars (^∗^*P <* 0.05; ^∗∗^*P <* 0.01; ^∗∗∗^*P <* 0.001). Data were compared by the Kruskal–Wallis test and Dunn multiple comparison post-test.

In order to elucidate the pregnancy outcome in the presence of *T. gondii* infection in both MIF^-/-^ and WT mice, we compared the profile of cytokines secreted in the serum of these animals. Concerning the Th1 profile of proinflammatory cytokines, IFN-γ and TNF secretion were higher in infected females, regardless of pregnancy, than in non-infected ones from both MIF^-/-^ and WT groups (*P <* 0.05) (**Figures 3A,B**). Higher levels of IFN-γ and TNF were detected in pregnant/infected WT females than MIF^-/-^ ones (*P <* 0.05) (**Figures 3A,B**). Conversely, pregnant/non-infected MIF^-/-^ females presented higher levels of IFN-γ when compared with their WT counterparts (*P <* 0.05) (**Figure 3A**). Analyses of IL-6 demonstrated a profile similar to that observed for IFN-γ and TNF, with detection of higher levels of IL-6 in pregnant/infected WT females than MIF^-/-^ ones (*P <* 0.05). WT infected females, pregnant or non-pregnant, presented higher secretion of IL-6 than non-infected ones (*P <* 0.001). In the MIF^-/-^ group, the increase in IL-6 secretion, due to infection, was detected only in the non-pregnant mice (*P <* 0.01), (**Figure 3C**).

On the other hand, analyses of the Th2 (IL-4, IL-2, and IL-10) and Th17 (IL-17) profiles of cytokines showed greater increase in the secretion in MIF^-/-^ females compared with WT ones (**Figures 3D–G**). Concerning IL-4, higher levels of this cytokine were detected in pregnant/non-infected MIF^-/-^ mice compared with WT (*P <* 0.001) (**Figure 3D**). In addition, IL-4 secretion was higher in pregnant/non-infected MIF^-/-^ mice when compared with pregnant/infected ones (*P <* 0.01) or when compared with non-pregnant/non-infected females of the same group (*P <* 0.001) (**Figure 3D**). No statistic difference were reached when IL-2 production where compared between WT and MIF^-/-^, however, MIF^-/-^ non-pregnant/infected mice presented tendency in higher IL-2 production compared with WT ones (*P* = 0.13, non significant). IL-10 and IL-17 presented similar results, that is, higher levels of these cytokines in MIF^-/-^ pregnant/non infected mice compared with WT (*P <* 0.01). Analyses of IL-10 and IL-17 in non-infected MIF^-/-^ group demonstrated higher levels of these cytokines in pregnant mice when compared with non-pregnant ones (*P <* 0.01), (**Figures 3D–G**).

### IL-4 Expression Is Upregulated in the Pregnant Uterus of Infected MIF^-/-^ Mice

**FIGURE 4:**
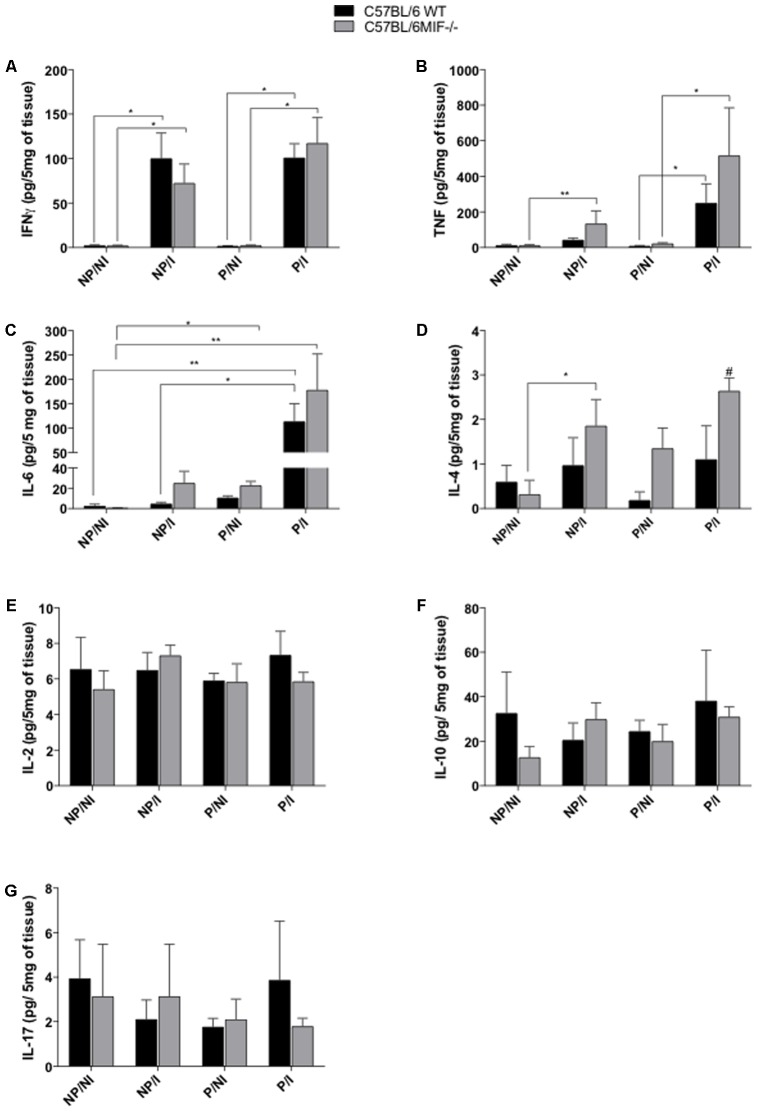
Cytometric bead assay (CBA) for cytokine measurements in uterus tissues from pregnant and non-pregnant C57BL/6 MIF^-/-^ and C57BL/6 WT mice orally infected with *Toxoplasma gondii.* Uterus tissue samples were collected within 8 days of infection/pregnancy. Uterus tissues from non-infected pregnant and non-pregnant mice were used as controls (*n* = 5 animals per group). The uterus tissue homogenates were prepared in a concentration of 200 mg/mL. Data are expressed as mean and SEM of the amount of cytokines (pg) present in 5 mg of tissue (pg/5 mg of tissue). **(A)** IFN-γ, **(B)** TNF-α, **(C)** IL-6, **(D)** IL-4, **(E)** IL-2, **(F)** IL-10, and **(G)** IL-17. ^#^Significant differences between MIF^-/-^ and WT mice in the same experimental condition (^#^*P <* 0.05). ^∗^Comparisons between multiple experimental conditions within the same group (NP/NI, non-pregnant/non-infected; NP/I, non-pregnant/infected; P/NI, pregnant/non-infected; P/I, pregnant/infected) are indicated by bars (^∗^*P <* 0.05; ^∗∗^*P <* 0.01; ^∗∗∗^*P <* 0.001). Data were compared by the Kruskal–Wallis test and Dunn’s multiple comparison post-test.

We also analyzed the cytokine profile at the maternal–fetal interface and uterus of non-pregnant females in order to investigate the outcome of *T. gondii* infection during pregnancy. Again, our results demonstrated an upregulation of Th1 (IFN-γ, TNF, and IL-6) cytokines due to infection, significantly for IFN-γ in both MIF^-/-^ and WT groups, regardless of pregnancy (*P <* 0.05) (**Figure 4A**). The expression of TNF was increased due to infection in the WT group, only in pregnant females (*P <* 0.01), whereas in the MIF^-/-^ group, infection induced increased TNF expression in both pregnant and non-pregnant females (*P <* 0.05) (**Figure 4B**). The production of IL-6 showed a profile similar to that of TNF, with upregulation of IL-6 induced by infection in the WT group only in pregnant females. In the infected WT group, IL-6 expression was higher in pregnant than in non-pregnant females (*P <* 0.01). In infected MIF^-/-^ females, infection induced IL-6 upregulation only in non-pregnant females (*P <* 0.05). Interestingly, in non-infected MIF^-/-^ mice, IL-6 expression was higher in pregnant than in non-pregnant females (*P <* 0.05) (**Figure 4C**). No statistically significant difference was obtained for Th1 cytokines when MIF^-/-^ and WT groups were compared.

Regarding the Th2 cytokine profile, IL-4 expression was apparently increased because of infection in both groups, but this upregulation was statistically significant only in infected/non-pregnant MIF^-/-^ females (*P <* 0.05). Also, IL-4 expression was more intensive in infected/pregnant MIF^-/-^ females than their WT counterparts (*P <* 0.05) (**Figure 4D**). No statistically significant differences were obtained for IL-2, IL-10, and IL-17 expression (**Figures 4E–G**, respectively).

### Indoleamine 2,3-Dioxygenase (IDO) Expression Is Upregulated at the Maternal–Fetal Interface of MIF^-/-^ Mice

**FIGURE 5:**
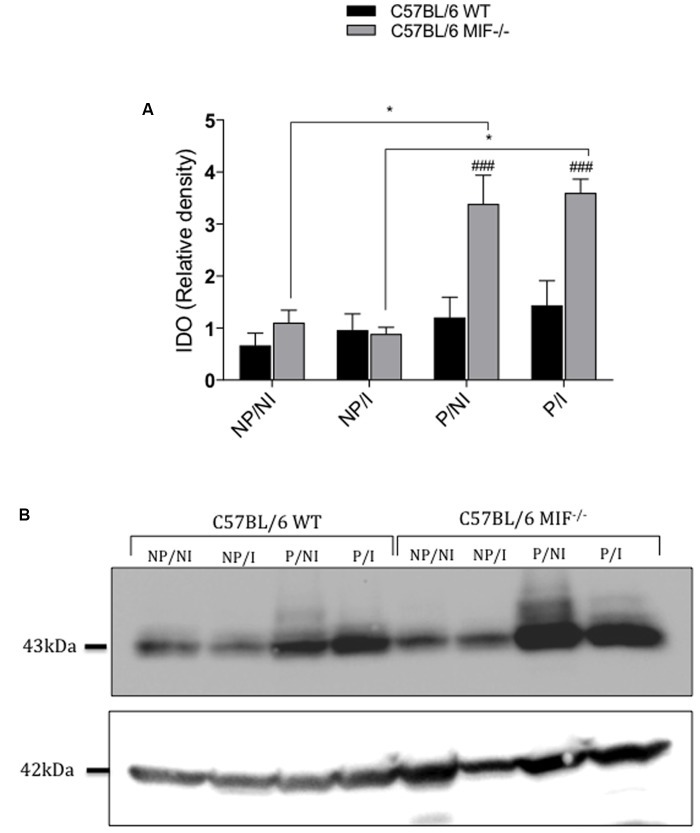
Indoleamine 2,3-dioxigenase (IDO) expression in uterus homogenates from pregnant and non-pregnant C57BL/6 MIF^-/-^ and C57BL/6 WT mice orally infected with *Toxoplasma gondii.* Uterus tissue samples were collected within 8 days of infection/pregnancy. Uterus tissues from non-infected pregnant and non-pregnant mice were used as controls. **(A)** IDO densitometric analyses are expressed as the relative density of the ratio between IDO and β-actin bands. Data are presented as mean and SEM of uterus samples (*n* = 5 animals per group). ^#^Significant differences between MIF^-/-^ and WT mice in the same experimental condition (^###^ *P <* 0.001). ^∗^Comparisons between multiple experimental conditions within the same group (NP/NI, non-pregnant/non-infected; NP/I, non-pregnant/infected; P/NI, pregnant/non-infected; P/I, pregnant/infected) are indicated by bars (^∗^*P <* 0.05). Data were compared by the Kruskal–Wallis test and Dunns’ multiple comparison post-test. **(B)** Representative cropped blots for IDO (43 kDa) and β-actin (42 kDa). All sample in the same gel were run simultaneously. Blots were probed with mouse anti-IDO and then stripped and reprobed with mouse anti β-actin, as a load control.

Next, we measured IDO expression in the uterus of pregnant and non-pregnant females, infected or not by *T. gondii*, in both MIF^-/-^ and WT groups. Our results clearly demonstrated that pregnant MIF^-/-^ females, regardless of infection, expressed more IDO at the maternal–fetal interface than its WT counterparts (*P <* 0.001). Also, IDO expression was upregulated in pregnant relative to non-pregnant MIF^-/-^ females, regardless of infection (*P <* 0.05) (**Figures 5A,B**).

### More Mast Cells (MC) Are Recruited in Tissues of Pregnant MIF^-/-^ Mice

**FIGURE 6:**
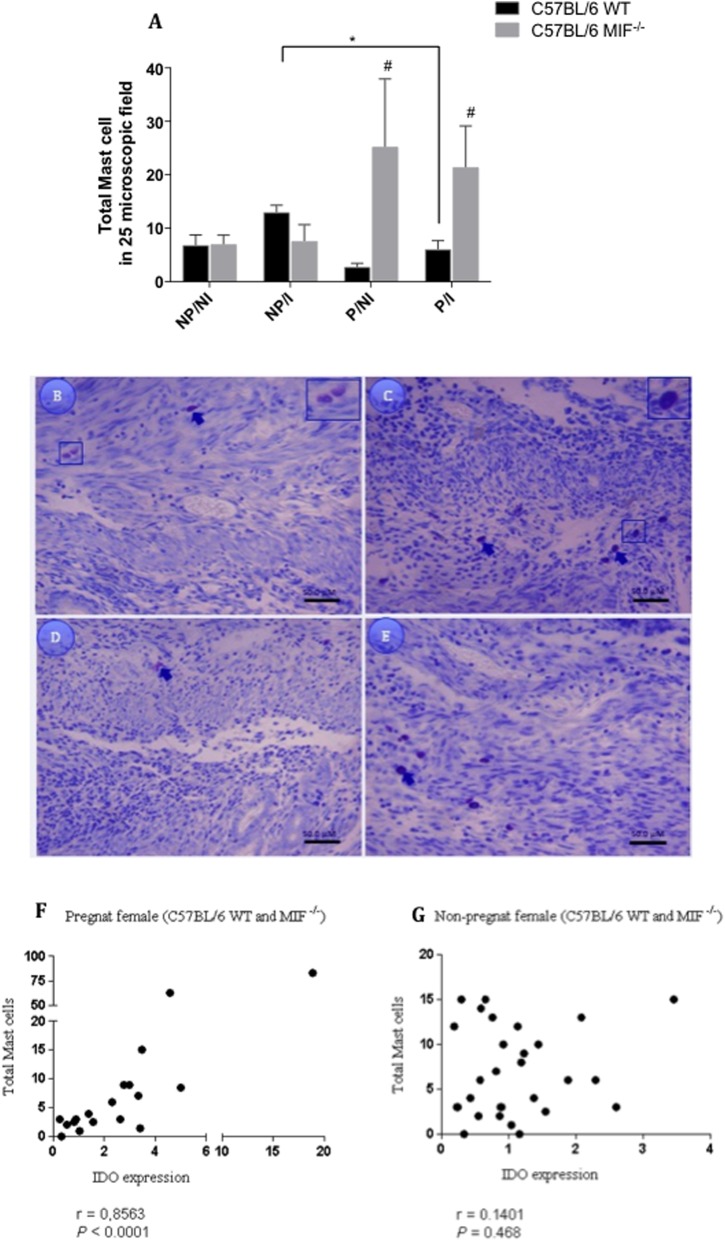
**(A)** Mast cell (MC) quantification in uterus/implantation sites from non-pregnant and pregnant females of C57BL/6 MIF^-/-^ and C57BL/6 WT mice infected or not by *Toxoplasma gondii* (*n* = 5 animals per group). Paraffin-embedded 4-μm tissue sections were stained with toluidine blue for MC quantification. MCs were counted in 25-microscopic fields per tissue section, and each animal was analyzed in two tissue sections. ^#^Significant differences between MIF^-/-^ and WT mice in the same experimental condition (^#^*P <* 0.05). ^∗^Comparisons between multiple experimental conditions within the same group (NP/NI, non-pregnant/non-infected; NP/I, non-pregnant/infected; P/NI, pregnant/non-infected; P/I, pregnant/infected) are indicated by bars (^∗^*P <* 0.05). Data were compared by the Kruskal–Wallis test and Dunn’s multiple comparison post-test. **(B–E)** Representative images from uterus tissues of pregnant/non-infected WT **(B)** and MIF^-/-^ **(C)** females; and pregnant/infected WT **(D)** and MIF^-/-^ **(E)** females. Arrows indicate granulated and degranulated MCs. Bar scale = 50 μM. Correlation between IDO expression and total MCs counted in the uterus of pregnant **(F)** and non-pregnant **(G)** C57BL/6 MIF^-/-^ and WT females.

Subsequently, we investigated the amount of MC in the uterus and implantation sites of pregnant and non-pregnant females, infected or not by *T. gondii*. Our results revealed that pregnant MIF^-/-^ females presented higher amount of MC compared with pregnant WT ones, regardless of infection (*P <* 0.05) (**Figure 6A**). In the infected WT group, we detected that non-pregnant females presented a higher amount of MC compared with pregnant females (*P <* 0.05) (**Figure 6A**). In all analyzed tissues, we detected both granulated and degranulated mast cells, with a higher frequency of granulated cells, but all of them were counted as total amount of MCs (**Figures 6B–E**). We also investigated the correlation between the total amount of MC and IDO expression in uterine tissues from both pregnant and non-pregnant MIF^-/-^ and WT females. Our results demonstrated a strong positive correlation between IDO expression and total MC counting (*r* = 0.8563, *P <* 0.0001 (**Figure 6F**) in pregnant females, whereas no correlation was found in non-pregnant ones (**Figure 6G**).

### MIF Is Upregulated in the Uterus of Infected WT Mice, and MIF Receptor (CD74) Is Upregulated at the Maternal–Fetal Interface of MIF^-/-^ Mice

**FIGURE 7:**
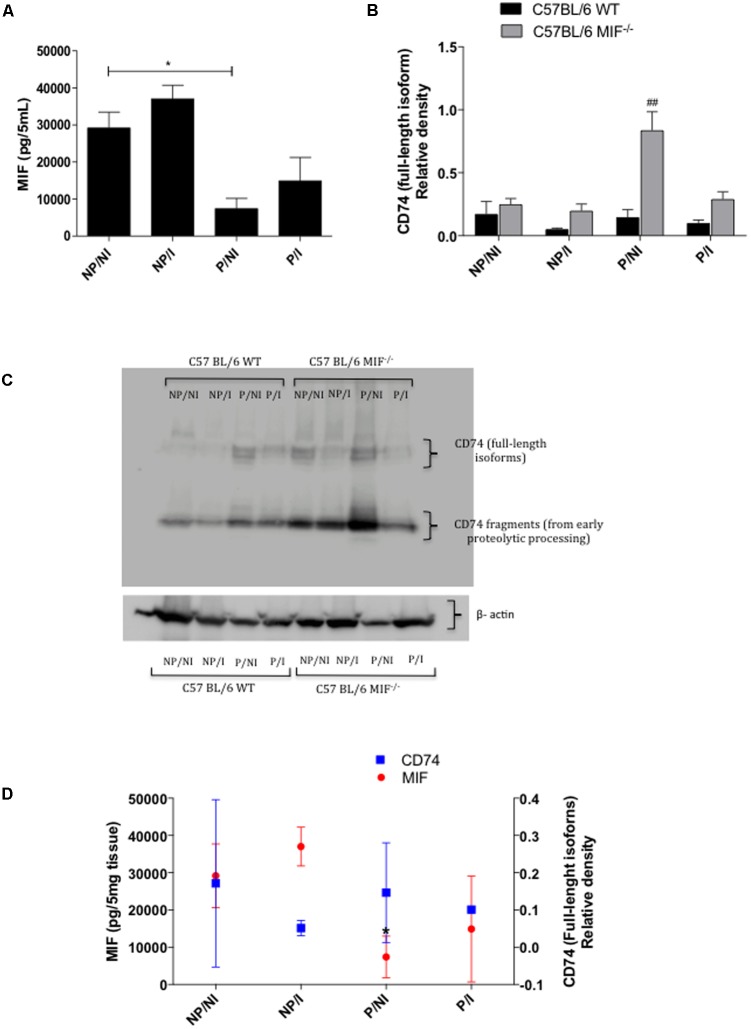
**(A)** ELISA for measurement of MIF in uterus homogenates of pregnant and non-pregnant C57BL/6 WT mice orally infected by *Toxoplasma gondii* (*n* = 5 animals per group). Uterus tissue samples were collected within 8 days of infection/pregnancy. Uterus tissues from non-infected pregnant and non-pregnant mice were used as controls. The homogenates were prepared in a concentration of 200 mg/mL. Data are expressed as the amount of cytokines (pg) present in 5 mg of tissue (pg/5 mg of tissue). NP/NI, non-pregnant/non-infected; NP/I, non-pregnant/infected; P/NI, pregnant/non-infected; P/I, pregnant/infected. **(B)** Relative density of CD74 expression (full-length isoforms) in the uterus homogenates from pregnant and non-pregnant MIF^-/-^ and WT females infected or not by *T. gondii.* Densitometric analyses are expressed as relative density of the ratio between CD74 and β-actin bands. Data are presented as mean and SEM of uterus samples from five animals per group. ^#^Significant differences between MIF^-/-^ and WT in the same experimental condition (^##^*P <* 0.01). ^∗^Comparisons between multiple experimental conditions within the same group (NP/NI, non-pregnant/non-infected; NP/I, non-pregnant/infected; P/NI, pregnant/non-infected; P/I, pregnant/infected) are indicated by bars (^∗^*P <* 0.05). Data were compared by the Kruskal–Wallis test and Dunn’s multiple comparison post-test. **(C)** Representative blot for CD74 (full-length isoforms and fragments) and cropped blot for β-actin (42 kDa). All sample in the same gel were run simultaneously. Blots were probed with mouse anti-CD74 and then stripped and reprobed with mouse anti β-actin as a loading control. Full-length blots are reported. **(D)** Association between MIF levels and CD74 (full-length isoforms) expression in uterus homogenates from pregnant and non-pregnant C57BL/6 WT mice orally infected with *T. gondii.* Uterus tissues from non-infected pregnant and non-pregnant mice were used as controls. ^∗^Asterisks indicate significant differences for MIF levels between non-infected pregnant and non-pregnant females (^∗^*P <* 0.05). Data were compared by the Kruskal–Wallis test and Dunn’s multiple comparison post-test.

In order to reinforce the role of MIF in the pregnancy outcome and *T. gondii* infection, we analyzed the MIF levels in uterus homogenates from WT mice. Our results demonstrated that MIF was slightly upregulated by infection in both pregnant and non-pregnant females, although statistical significance was not reached. Also, MIF levels were decreased significantly in pregnant females relative to non-infected ones (*P <* 0.05) (**Figure 7A**). Next, we investigated MIF receptor (CD74) expression in the uterus of both MIF^-/-^ and WT mice. Our results demonstrated that CD74 (full-length isoforms) expression was higher in pregnant MIF^-/-^ females than in their WT counterparts, regardless of infection. This difference was statistically significant for non-infected pregnant females (*P <* 0.01) (**Figures 7B,C**). Our results also demonstrated that MIF^-/-^ females presented increased expression of the CD74 fragments coming from early proteolytic processing (**Figure 7C**). Subsequently, we investigated whether CD74 expression and MIF levels in the uterus from WT mice could be related. Our results demonstrated that an increase in MIF expression due to infection was followed by a decrease in CD74 expression in the uterus from both non-pregnant and pregnant WT females (**Figure 7D**).

### Parasitism Is Higher in MIF^-/-^ Mice Than in Their WT Counterparts

**FIGURE 8:**
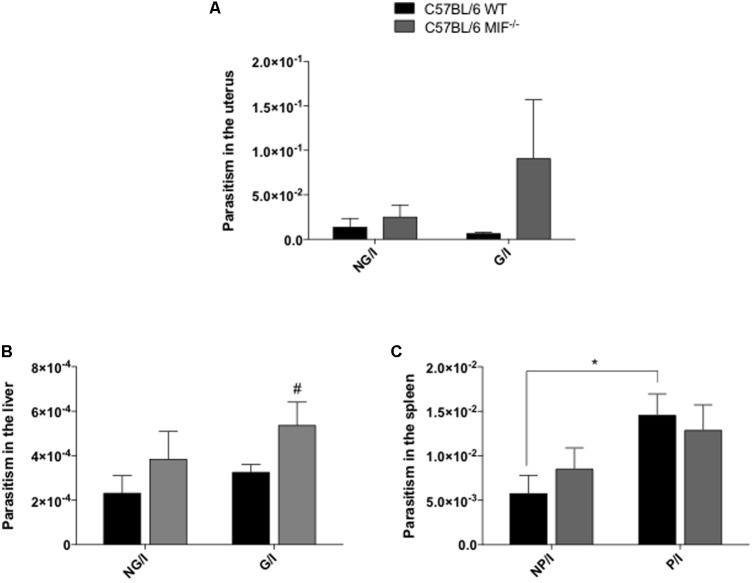
Parasite burden in the **(A)** uterus, **(B)** liver, and **(C)** spleen from non-pregnant and pregnant WT and MIF^-/-^ mice quantified by real-time PCR. Females were orally infected with five cysts of *Toxoplasma gondii* and euthanized with 8 days of infection/pregnancy. Data are expressed as mean and SEM values obtained from five animals per group. Data were compared by the Kruskal–Wallis test and Dunn’s multiple comparison post-test. ^#^Significant differences between MIF^-/-^ and WT in the same experimental condition (^#^ *P <* 0.01). ^∗^Comparisons between multiple experimental conditions within the same group (NP/I, non-pregnant/infected; P/I, pregnant/infected) are indicated by bars (^∗^*P <* 0.05).

Finally, we assessed the parasitism in the uterus, liver, and spleen from pregnant and non-pregnant MIF^-/-^ and WT females infected with five cysts of *T. gondii*. Our results demonstrated a tendency for increasing parasitism in the pregnant uteri (*P* = 0.24, not significant), (**Figure 8A**) and significant increase parasitism in the livers of pregnant MIF^-/-^ females compared with WT ones (*P <* 0.05), (**Figure 8B**). In non-pregnant females, there was a slightly increase in parasitism in all organs analyzed from MIF^-/-^ mice compared with WT, but the difference was not significant (**Figures 8A–C**). Overall, when pregnant and non-pregnant females were compared, higher tissue parasitism was detected in pregnant than non-pregnant females of both groups, but with statistically significant differences only in the spleen from WT females (*P <* 0.05) (**Figure 8C**).

## Discussion

Congenital toxoplasmosis is still considered a public health problem worldwide. When *T. gondii* infection takes place during pregnancy, transplacental transmission can occur (Li et al., 2014) and can lead to the development of specific symptoms in neonates, such as retinochoroiditis, intracranial calcifications, hydrocephalus, and neurological disabilities (Maenz et al., 2014). Additionally, congenital toxoplasmosis can induce abortion and preterm labor, depending on the gestational age when infection occurs (Robbins et al., 2012). Our group has investigated the role of MIF in congenital toxoplasmosis, since MIF is constitutively expressed during pregnancy and possibly associated with the physiology of gestation (Bevilacqua et al., 2014). Additionally, MIF plays an important role in the host defense against infection by parasites (Rosado and Rodriguez-Sosa, 2001). On the other hand, MIF has also been associated with numerous pathologies. For example, the control of infection mediated by MIF is associated with increased systemic inflammation, tissue damage, and death (Cavalcanti et al., 2011). Also, the role of MIF in inducing cell proliferation and apoptosis inhibition makes it an important target for anticancer therapies (Guo et al., 2013). Accordingly, MIF has been demonstrated to be associated with both protective and deleterious roles; therefore, therapeutic approaches have been tried based on MIF roles (Bloom et al., 2016). Considering these controversial activities of MIF, new studies are required to clarify the potential roles of MIF. In that sense, knockout mice have been employed as a useful tool in cell biology (Nelson, 1997). Therefore, we have proposed herein the MIF knockout C57BL/6 mice as a study model to investigate MIF function in congenital toxoplasmosis.

Initially, we observed that non-pregnant MIF^-/-^ mice lost more body weight in response to *T. gondii* infection compared with WT mice. Likewise, pregnant MIF^-/-^ females succumbed to oral infection by *T. gondii* before WT ones. These mortality data agree with a previous study that demonstrated the role of MIF in the resistance of non-pregnant BALB/c mice to *T. gondii* infection, since BALB/c MIF^-/-^ mice presented a higher mortality rate than their WT counterparts in response to oral infection (Terrazas et al., 2010). BALB/c MIF^-/-^ mice also presented increased mortality rates due to intraperitoneal infection with the highly virulent RH strain (Flores et al., 2008). In our study, pregnant MIF^-/-^ females belonged to the C57BL/6 genetic background, which is naturally more susceptible to *T. gondii* infection than BALB/c (Coutinho et al., 2012). Previous studies demonstrated that C57BL/6 mice infected by *T. gondii* presented a severe Th1-type immunopathology and important susceptibility to infection despite of the type-1 immune response (Liesenfeld, 2002). Conversely, in the absence of endogenous IFN-γ, which is a key cytokine of the Th1 profile, mice rapidly succumb to infection because of unchecked tachyzoite replication (Scharton-Kersten et al., 1996). Thus, we investigated the immune response, pathology, tissue parasitism and expression of regulatory proteins in MIF^-/-^ mice infected with *T. gondii* during pregnancy in order to clarify our mortality results.

Concerning pregnancy outcome, we found important damage in both MIF^-/-^ and WT mice due to *T. gondii* infection. However, infected MIF^-/-^ females presented fewer reabsorption sites and, consequently, a higher percentage of normal implantation sites when compared with WT ones. These data match the pathology results, demonstrating that necrotic and hemorrhagic findings were more common in WT females. Thus, the pregnancy outcome was less damaging for MIF^-/-^ mice compared with WT ones, considering 8 days of pregnancy/infection. However, all analyzed mice presented abortion after this period, because none pregnant mice, that was followed during 80 days for mortality study, gave birth during this period. Our data are in agreement with a previous study, using C57BL/6 pregnant mice, which demonstrated that, at 8 days of pregnancy/infection, 100% of the analyzed mice presented implantation sites. However, at 19 days of pregnancy/infection, 90% of the animals had abortion (Coutinho et al., 2012). Thus, based on our results, it is possible to suggest that absence of MIF contributed to reduction in the damage caused by *T. gondii* infection during pregnancy, but it was not sufficient to prevent abortion that occurred later.

Next, we analyzed the systemic and local cytokine profiles of these animals. Pregnant and non-pregnant females from both WT and MIF^-/-^ groups presented a robust systemic Th1-type immune response to *T. gondii* infection, but levels of serum proinflammatory cytokines, such as TNF and IFN-γ, were higher in the WT group. These results agree with MIF functions, since MIF modulates the expression of inflammatory molecules, such as TNF, nitric oxide, and cyclooxygenase 2 (COX-2) (Rosado and Rodriguez-Sosa, 2001). The absence of MIF in pregnant mice was associated with increase in serum levels of Th2/T regulatory (Treg) cytokines (IL-4 and IL-10). Inflammation has been associated with negative pregnancy outcomes, such as, abortion, preterm birth and pre-eclampsia. On the other hand, a healthy pregnancy is mostly associated with Th2-type immune response (Romero et al., 2007). Although, some events of the pregnancy such as implantation, placentation and parturition are associated with pro-inflammatory cytokines, the imbalance in these immune response triggered by pathogens is often associated with worst pregnancy outcome (Mor et al., 2017). In the present study, pregnant MIF^-/-^ mice presented higher level of IL-17 when compared with their WT counterparts. The increase in IL-17 secretion in the serum these animals can be a compensatory mechanism for MIF absence. In this sense, the increase of IL-10 (whose secretion profile was similar to that obtained for IL-17), may have occurred as an attempt to maintain the fine balance between Th17/Treg profiles. Th17 and Treg cells are important players in the establishment of defense and tolerance (Figueiredo and Schumacher, 2016). The imbalance in the number of Treg and Th17 cells and related cytokines, triggered by infections, is associated to embryo loss (Zhang et al., 2012).

In the present study, cytokine production at the maternal–fetal interface reflects the systemic levels, showing an important and similar Th1-type profile in response to infection in both WT and MIF^-/-^ groups. Regarding the Th2-type cytokine profile, IL-4 was upregulated at the maternal–fetal interface from MIF^-/-^ females. These results support our pregnancy outcome data, suggesting that modulation of the immune response in the absence of MIF was favorable to the early phase of pregnancy. Coutinho et al. (2012) found similar results when comparing pregnancy outcome in C57BL/6 and BALB/c mice infected by *T. gondii*, attributing the worst result of pregnancy in C57BL/6 mice to a strong proinflammatory immune response (Coutinho et al., 2012). Studies using the mouse model suggest that production of more Th2 than Th1 cytokines by maternal cells prevents fetal rejection (Lin et al., 1993). Likewise, successful pregnancy can be achieved by a subset of epigenetically modified maternal immune cells that can create a tolerogenic niche for the development of the semiallogeneic fetus (Arck and Hecher, 2013). Additionally, high levels of IFN-γ were related to fetal reabsorption at the implantation sites in murine models of *T. gondii* infection (Ge et al., 2008), whereas the lack of IFN-γ was related to maternal–fetal transmission of *T. gondii* (Shirahata et al., 1992; Shiono et al., 2007). Altogether, these data reinforce the association between cytokine production and the reabsorption rates found in our study.

In this survey, IL-6 was also increased in infected animals. We also found that systemic IL-6 was more pronounced in the WT group. Interleukin-6 has been described as a pivotal cytokine that exerts regulatory functions in embryo implantation and placental development. However, at elevated levels, IL-6 potentially inhibits the generation of CD4+ Treg cells required for pregnancy tolerance (Prins et al., 2012). Although proinflammatory cytokines are important for some gestational events, such as implantation and vascularization, it is equally important to maintain an adequate equilibrium in these cytokine levels, since the imbalance is associated with gestational loss (Kalagiri et al., 2016).

To test our hypothesis that the differences in pregnancy outcome between MIF^-/-^ and WT mice could be associated with their immunologic profiles, we assessed IDO expression at the maternal–fetal interface. Our results showed that IDO is upregulated in pregnant MIF^-/-^ mice, regardless of *T. gondii* infection. To the best of our knowledge, this is the first time that the absence of MIF was associated with increased IDO expression. IDO has been proposed as an important catabolizer of the amino acid tryptophan, which plays an important role in the suppression of T-cell proliferation and consequently, gestational success (Munn et al., 1998). IDO catabolizer function also presents antimicrobial properties, being capable of inhibiting the growth parasites, such as, *T. gondii* (Schmidt et al., 2009). Additionally, allogeneic fetuses exposed to IDO inhibitor (1-methyl-tryptophan) present complement activation, inflammation, and fetal rejection (Mellor et al., 2001). Thus, in the present study, the worst pregnancy outcome found in WT mice might be associated with IDO inhibition due to MIF activity. In this sense, decreased IDO expression can lead to pronounced proliferation of proinflammatory T cells responsible for the imbalance in proinflammatory cytokines that causes damage during gestation. On the other hand, increase in IDO expression, in MIF^-/-^ pregnant mice, was not associated with control of infection in the uterus these animals. Probably, the proliferation in T cell and production of inflammatory cytokines were decisive for controlling of infection in WT mice.

After detecting increased IDO expression in pregnant MIF^-/-^ mice, we analyzed the presence of MC at the maternal–fetal interface. MCs have been reported as important cells able to interact with pathogens and allergens, having decisive roles in promoting inflammation and modulation of the immune response (Nakae et al., 2006). Likewise, our results showed that MCs were more concentrated in tissues of pregnant MIF^-/-^ females, regardless of *T. gondii* infection. In contrast, a previous study showed that MIF promotes recruitment of MC in a manner that depends on transducer and activator of transcription 5 (STAT-5) (Põlajeva et al., 2014). These authors also demonstrated that antibodies that neutralize MIF could attenuate MC infiltration. In fact, our results showed a higher number of MC in non-pregnant WT than MIF^-/-^ mice, although the difference was not significant, whereas pregnant MIF^-/-^ mice recruited more MC than their WT counterparts, whether infected or not. Interestingly, pregnant females that presented a higher number of MC were the same ones that presented higher IDO expression, resulting in an important positive correlation between these parameters. A previous study demonstrated that MCs together with regulatory T cells (Tregs) are able to induce immune tolerance in mice (de Vries et al., 2011). The mechanism of immune tolerance mediated by MCs is associated with an increase in IDO expression by immature DCs. DCs are important to create a tolerogenic environment in the maternal–fetal interface, stimulating the growth and maintenance of regulatory T cells due to contact with MCs (Rodrigues et al., 2016). Altogether, the reduction of injury in the maternal–fetal interface from *T. gondii-*infected MIF^-/-^ mice can be associated with the increased recruitment of MCs that potentiate IDO expression in these tissues, leading to a tolerogenic profile, probably due to polarization of T lymphocytes toward a regulatory profile.

To investigate if MIF is involved in pathology, we measured MIF levels in the uterus of pregnant and non-pregnant WT mice, infected or not by *T. gondii*. A slight increase in MIF levels due to infection was seen in both pregnant and non-pregnant females. MIF upregulation because of *T. gondii* infection was previously described in our group in human placental explants, being associated with the control of infection in these tissues (Ferro et al., 2008). Finally, we investigated the MIF receptor (CD74) expression in MIF^-/-^ and WT mice. Surprisingly, an important increase in CD74 expression, both the full-length isoform and fragments, coming from early proteolytic processing, was found in pregnant MIF^-/-^ females. Also, CD74 expression showed an inverse association with MIF expression in the uteri of WT mice.

Migration inhibitory factor activity depends on its interaction with the receptor present on the cell surface. Interaction between MIF and CD74 causes activation of the MAP kinase signaling pathways and a further response that includes activation of transcription factors leading to cell proliferation, survival, and production of inflammatory mediators (Calandra and Roger, 2003). CD74 also plays a well-established role in antigen presentation that depends on proteolytic cleavage in the endosomal compartments (Bergmann et al., 2013).

It is not entirely clear to us why the MIF receptor was upregulated in MIF^-/-^ mice in the present study. One possibility would be a compensatory mechanism for MIF deficiency. New studies should be conducted to investigate if another molecule could bind to the upregulated receptor as a compensatory mechanism in these knockout animals. D-dopachrome tautomerase (D-DT) was described as a MIF-homolog that can bind to CD74 with high affinity, leading to activation of MAP kinase signaling pathways and proinflammatory activities (Merk et al., 2011). Furthermore, in *T. gondii*-infected females, it is possible that CD74 can be activated by MIF from *T. gondii* (*Tg*MIF). It was previously described that *Tg*MIF presented structural homology with mammalian MIFs being able to activate the ERK/MAPK pathways in murine macrophages (Sommerville et al., 2013). If D-DT or *Tg*MIF binds to the upregulated MIF receptor in MIF^-/-^ females, it is possible that MIF activities are not completely abrogated. This hypothesis was supported by our gestational results: although the damage was reduced in MIF^-/-^ mice, it was not completely abolished. Further studies are required to verify if, when congenital toxoplasmosis is studied in a system completely deficient in MIF, the pathology and parasitism will be importantly modified.

Although the pathology data could support the pregnancy outcome, it could not explain the results of mortality in MIF^-/-^ mice. Thus, we investigated tissue parasitism at the maternal–fetal interface and maternal (liver and spleen) tissues. The majority of the analyzed tissues presented a higher parasitism index in MIF^-/-^ females than WT ones, reinforcing data from the literature indicating that MIF is an important factor in parasitism control because of the inflammatory immune response that is associated with MIF (Flores et al., 2008; Terrazas et al., 2010; de Oliveira Gomes et al., 2011). These results of mortality could be associated with uncontrolled tachyzoite replication. Cavalcanti et al. (2011) suggested that MIF controls *T. gondii* infection in susceptible mice, but at the expenses of increased local and systemic inflammation, tissue damage, and death (Cavalcanti et al., 2011). Our data suggest that absence of MIF was associated with a better pregnancy outcome at the expenses of the maternal life due to increased parasite replication.

**FIGURE 9:**
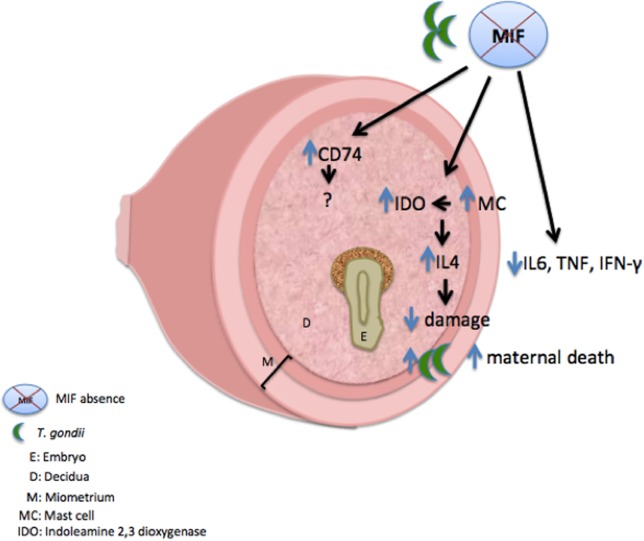
Proposed scheme for the immune response mechanism to congenital toxoplasmosis in C57BL/6 MIF^-/-^ females. C57BL/6 MIF^-/-^ pregnant females (8 dop) infected by *T. gondii* presented upregulation in MIF receptor (CD74), as a possible compensatory mechanism for MIF absence. Additionally, these mice presented increased MC recruitment at the maternal fetal interface that can be associated with increased IDO expression at this interface. IDO upregulation is probably associated with increased IL-4 expression in these tissues. Likewise, C57BL/6 MIF^-/-^ females presented a decrease in systemic proinflammatory cytokines. Altogether, these data clarify that the effects on pregnancy outcome are less harmful in C57BL/6 MIF^-/-^ females but still indicate *T. gondii* proliferation and early death in these animals.

In conclusion, MIF has been shown to be an important factor that is expressed at the maternal–fetal interface in response to *T. gondii* infection, controlling infection but inducing abortion and pathology. MIF pathology at the maternal–fetal interface can be associated with low recruitment of MC and downregulation in IDO expression and consequent upregulation of the proinflammatory immune response (**Figure 9**). C57BL/6 MIF^-/-^ mice can be used as a good model for studying MIF function at the maternal–fetal interface, since redundant or compensatory mechanisms, such as increased receptor expression, are considered, and this result can reinforce the importance of MIF in the maternal-fetal interface.

## Materials and Methods

### Mice and Mating

All experiments were carried out with female mice (8–12 weeks old) genetically deficient in *Mif* (MIF^-/-^) on a C57BL/6 genetic background and age and sex-matched C57BL/6 WT mice. C57BL/6 MIF^-/-^ mice were kindly provided by professor Dr. Marcelo Torres Bozza from the Federal University of Rio de Janeiro, Brazil. MIF^-/-^ mice generation was performed as previously described (Bozza et al., 1999). Briefly, a *Mif* disrupted genomic vector was transfected by electroporation into 107 J1 embryonic stem (ES) cells. Clones were obtained by homologous recombination and than, injected into C57BL/ 6 blastocysts. Next, blastocysts were transferred into pseudopregnant females and chimeric mice were bred with C57BL/ 6 mice for reaching agouti offspring. The animals were maintained under standard conditions in the Bioterism Center and Animal Experimentation of the Federal University of Uberlândia, Brazil, on a 12 h light/dark cycle and with free access to food and water. The study was approved by the Animal Experimental Ethics Committee (CEUA) of the Institution under protocol number 002/09. All experiments were performed in accordance with Brazilian guidelines involving animal use in research. For mating, one male and two females of the same genetic background were housed overnight in the same cage. Females were checked every morning for the presence of vaginal plugs (mix of sperm and vaginal secretion). Plug detection was used as indicator of the first day of pregnancy.

### Experimental Infection

*Toxoplasma gondii* ME-49 strain cysts were gathered from the brains of *Calomys callosus* (Rodentia: Cricetidae) previously inoculated with 20 cysts. C57BL/6 MIF^-/-^ and WT mice were orally infected on the first day of the pregnancy with five cysts of the ME-49 strain. Control groups consisted of non-infected pregnant females and non-pregnant females infected or not with five cysts of the ME-49 strain. Each experimental group comprised a minimum of five animals. Alternatively, non-pregnant females from both genetic backgrounds were infected with 10 cysts of the ME-49 strain and were followed to verify the change in the body weight during 21 days.

### Experimental Design

**FIGURE 10:**
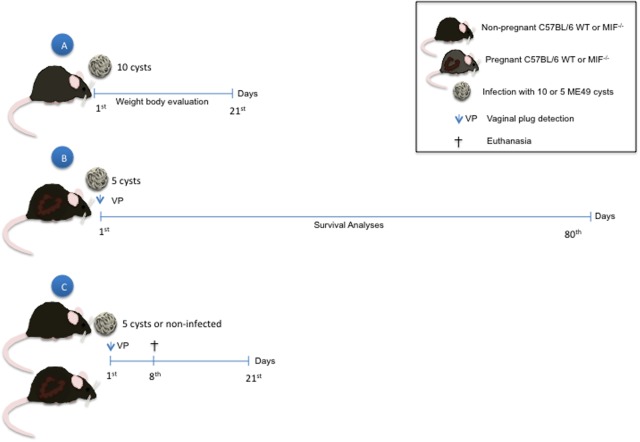
Experimental design: **(A)** Non-pregnant mice from both genetic backgrounds (WT and MIF^-/-^) were infected with 10 cysts of the ME-49 strain and were followed to verify the change in the body weight during 21 days. **(B)** Pregnant mice from both genetic backgrounds were infected with five cysts of the ME-49 strain on the day of detection of vaginal plug (vp), that is the first day of pregnancy (1st dop). Mice were followed during 80 days to survival analyses. **(C)** Pregnant and non-pregnant mice from both genetic backgrounds were infected with five cysts of the ME-49 strain on 1st dop. Animals were euthanized on day 8 post-infection. Blood sample was collected for cytokines analyzes. Liver, spleen and uterus were processed for parasitism analyses by PCR. Uteri have also been anatomically analyzed for evaluation of implantation sites (presence of reabsorption, hemorrhage and necrosis). Alternatively, uteri were used for cytokine measurements, protein analyzes by western blotting (IDO and CD74), histopathological evaluation and mast cell quantification.

Pregnant and non-pregnant females were anesthetized on day 8 post-infection by intraperitoneal injection with ketamine (Syntec Brasil Ltd., Syntec Brasil Ltd, SP, Brazil) and xylazine (Schering-Plough Coopers, Cotia, SP, Brazil), and blood samples were collected by puncture of the retro-orbital plexus. Then, animals were euthanized by cervical dislocation, and organs (liver, spleen, and uterus) were collected for further parasite quantification by PCR. Serum samples were used for cytokine measurement by CBA. Uterine tissue samples were processed for cytokine quantification by CBA, western blotting, and histopathological analyses. Non-infected females were analyzed under the same conditions. Alternatively, non-pregnant females of both genetic backgrounds (MIF^-/-^ and WT) infected with 10 cysts of strain ME-49 were analyzed daily for changes in body weight for 21 days. Additionally, pregnant females infected with five cysts of strain ME-49 were followed for the mortality index (**Figure 10**).

### Cytokine Measurements in Uterine Tissue and Serum Samples

For intracellular cytokine quantification in the uterus, tissues were weighed and then homogenized in radioimmunoprecipitation assay buffer (RIPA) [50 mmol/L Tris hydrochloride, 150 mmol/L NaCl, 1% (v/v) Triton X-100, 1% (w/v) sodium deoxycholate, and 0.1% (w/v) SDS; pH 7.5] to which was added protease inhibitor cocktail (Complete^®^, Roche Diagnostic, Mannheim, Germany). The volume of the solution was adjusted according to the uterus weight to obtain a final concentration of 200 mg/mL. After centrifugation at 15,000 ×*g* for 20 min at 4°C, the supernatants were collected and used for quantification of intracellular cytokines by CBA.

Cytokines from Th1/Th2/Th17 profiles were measured in supernatants of uterine tissue extract and in serum samples of all experimental groups using a CBA kit (BD Bioscience, San Diego, CA, United States) according to the manufacturer’s instructions. The samples were analyzed by BD^TM^ flow cytometry (FACSCalibur, BD Company, San Diego, CA, United States). Data were recorded using specialized BD^TM^ Cell Quest software and expressed as pg/mL for serum samples and pg/5 mg of tissue for the uterus extract.

Alternatively, MIF were measured in 5 mg of uterus tissue extract using Mouse MIF DuoSet enzyme-linked immunosorbent assay (ELISA), (R&D Systems, Abingdon, Oxfordshire, England), according to the manufacturer’s recommendations. MIF concentration was determined via extrapolation from a standard curve obtained from known concentrations of mouse rMIF and data were expressed as pg/5 mg of tissue. The assay sensitivity was 18 pg/mL.

### Quantification of Parasitism by PCR

Total DNA was extracted from 20 mg of each tissue (uterus, spleen, and liver) from pregnant and non-pregnant MIF^-/-^ and WT females infected by *T. gondii*. The extraction was performed using the Wizard^®^ Genomic DNA Purification Kit (Promega Co., Madison, WI, United States), following the manufacturer’s instructions. The parasite load in the tissues was determined by qPCR using GoTaq^®^ qPCR Master Mix (Promega) targeting a region of 529 base pairs (bp) that repeats 200–300 times in the *T. gondii* genome using the following primers: forward 5′-CACAGAAGGGACAGAAGT-3′ and reverse 5′-TCGCCTTCATCTACAGTC-3′ (Edvinsson et al., 2006). The amplifications and analyses were performed in a Step One Plus^®^ Real Time PCR System (Applied Biosystems, Foster City, CA, United States). The threshold cycle (Ct) value for each sample was compared to the standard curve (10^3^ to 10^7^ parasites/mL) and the relative parasite concentration analyzed. *T. gondii*, ME-49 strain, tachyzoites were obtained from HeLa cell culture for preparing standard curve. Positive and negative parasite controls were included in each assay.

### Western Blotting

Uterus tissue samples from pregnant and non-pregnant C57BL/6 MIF^-/-^ and WT mice, infected or not by *T. gondii*, were homogenized in RIPA buffer as described above, and supernatants were used for protein quantification using the Bradford method (Bradford, 1976). Next, 100 μg of total protein was separated through 10% polycrylamide gel under denaturing conditions (SDS-PAGE) and transferred to PVDF membranes (Thermo Scientific). Membranes were blocked with 4% fat dry milk in 20 mM Tris-buffered saline, pH 7.2 (TBS) for 1 h at room temperature. Next, membranes were incubated overnight (4°C) with primary antibodies as rabbit anti-mouse CD74 (Santa Cruz Biotechnology, Dallas, TX, United States) or goat anti-mouse IDO (Santa Cruz Biotechnology) diluted in TBS plus 2% fat dry milk. The housekeeping control was performed by incubating the membranes with rabbit anti-mouse β-actin (Santa Cruz Biotechnology). Next, the membranes were incubated with the respective secondary antibodies conjugated with peroxidase (Jackson ImmunoResearch Laboratories, West Grove, PA, United States) diluted in TBS plus 2% fat dry milk for 2 h at room temperature. The reaction was developed by using a chemiluminescence kit (ECL kit SuperSignal, Thermo Fisher Scientific, Waltham, MA, United States). Densitometric analysis and membrane documentation were performed with a ChemiDoc MP Imaging System (BIO-RAD Laboratories Inc, Hercules, CA, United States), using Image Lab^TM^ software. Data are presented as the relative density of the bands obtained from the ratio between the protein of interest and β-actin bands.

### Histopathological Analysis and Mast Cell (MC) Quantification

The uterus was examined macroscopically for small-sized implantation sites or those with a necrotic or hemorrhagic appearance in order to determine the presence of reabsorption sites. For histopathological analyses, 4-μm tissue sections were stained with hematoxylin-eosin. The fragments were analyzed under light microscopy for the presence of inflammatory cell infiltrates and necrotic and hemorrhagic implantation sites.

Alternatively, 4-μm uterus tissue sections of pregnant and non-pregnant females were stained with 0.5% toluidine blue in citrate buffer in order to detect MC. For MC quantification, two sections, distant by at least 40 μm one from other, were used per animal. Next, 25 images were captured from each tissue section using a Nikon i50 microscope (40x objective) coupled to a capture image system. The images were obtained following a pattern of capture, in which, images were acquired from the marginal region of the tissue going to the center, that is, from the myometrium to the endometrium. The captures were obtained in an alternated way, in other words, for each microscopic field that was acquired, one field was disregarded. Both granulated and degranulated MCs were quantified, and the results were expressed as the mean of the total MC captured in 25 microscopic fields.

### Statistical Analysis

Data were analyzed using GraphPad Prism 7 (GraphPad Software Inc., San Diego, CA, United States). Data were expressed as mean ± SEM of experimental groups. Survival data were presented as a Kaplan–Meier survival curve and analyzed with a log-rank test. Data on histopathological analysis of the implantation sites are presented as descriptive analyses. The comparisons between C57BL/6 MIF^-/-^ and WT mice were analyzed by the Mann–Whitney test, whereas comparisons between different experimental conditions within each mouse lineage were analyzed by the Kruskal–Wallis test and Dunn multiple comparison post-test. Correlation between IDO and MC results was performed using the Pearson correlation coefficient. Differences were considered statistically significant at *P <* 0.05.

## Author Contributions

AG and EF designed the research. AG, BB, PF, MR, RS, PSG, KA, MA, PMG, and AC performed the research. JVC and JLC performed the Histomorphometric analysis on mast cell data. NS performed pathological analysis. AG analyzed the data and wrote the paper. TM, JM, and EF revised the paper and contributed with reagents and analytic tools.

## Conflict of Interest Statement

The authors declare that the research was conducted in the absence of any commercial or financial relationships that could be construed as a potential conflict of interest.
